# CD163^+^CD204^+^ tumor-associated macrophages contribute to T cell regulation via interleukin-10 and PD-L1 production in oral squamous cell carcinoma

**DOI:** 10.1038/s41598-017-01661-z

**Published:** 2017-05-11

**Authors:** Keigo Kubota, Masafumi Moriyama, Sachiko Furukawa, Haque A. S. M. Rafiul, Yasuyuki Maruse, Teppei Jinno, Akihiko Tanaka, Miho Ohta, Noriko Ishiguro, Masaaki Yamauchi, Mizuki Sakamoto, Takashi Maehara, Jun-Nosuke Hayashida, Shintaro Kawano, Tamotsu Kiyoshima, Seiji Nakamura

**Affiliations:** 1 Section of Oral and Maxillofacial Oncology, Division of Maxillofacial Diagnostic and Surgical Sciences, Faculty of Dental Science, Kyushu University, Fukuoka, 812-8582 Japan; 2 OBT Research Center, Faculty of Dental Science, Kyushu University, Fukuoka, 812-8582 Japan; 3 Laboratory of Oral Pathology, Division of Maxillofacial Diagnostic and Surgical Sciences, Faculty of Dental Science, Kyushu University, Fukuoka, 812-8582 Japan

## Abstract

Tumor-associated macrophages (TAMs) promote cancer cell proliferation, invasion, and metastasis by producing various mediators. Although preclinical studies demonstrated that TAMs preferentially express CD163 and CD204, the TAM subsets in oral squamous cell carcinoma (OSCC) remain unknown. In this study, we examined the expression and role of TAM subsets in OSCC. Forty-six patients with OSCC were analyzed for expression of TAMs in biopsy samples by immunohistochemistry. We examined TAM subsets and their production of immune suppressive molecules (IL-10 and PD-L1) in peripheral blood mononuclear cells from three OSCC patients by flow cytometry. CD163 was detected around the tumor or connective tissue, while CD204 was detected in/around the tumors. Flow cytometric analysis revealed that CD163^+^CD204^+^ TAMs strongly produced IL-10 and PD-L1 in comparison with CD163^+^CD204^−^ and CD163^−^CD204^+^ TAMs. Furthermore, the number of activated CD3^+^ T cells after co-culture with CD163^+^CD204^+^ TAMs was significantly lower than that after co-culture with other TAM subsets. In clinical findings, the number of CD163^+^CD204^+^ TAMs was negatively correlated with that of CD25^+^ cells and 5-year progression-free survival. These results suggest that CD163^+^CD204^+^ TAMs possibly play a key role in the invasion and metastasis of OSCC by T-cell regulation via IL-10 and PD-L1 production.

## Introduction

Monocytes/macrophages are important contributors to cancer-associated inflammation. The heterogeneity of macrophages has been discussed with regard to different responses to various microenvironmental stimuli. Macrophages are classified into two distinct subtypes: the classically activated (M1) macrophage stimulated by microbial products and interferon-γ, and the alternatively activated (M2) macrophage stimulated by IL-4, IL-13, and IL-10^1–4^. Several studies have shown that M2 macrophages infiltrating into the tumor microenvironment contribute to cancer progression and are associated with tumor progression, angiogenesis, metastasis and immunosuppression. This macrophage phenotype is referred to as the tumor-associated macrophage (TAM)^5–7^.

CD163 and CD204-positive macrophages are positively correlated with the histological gradient of malignancy in human ovarian tumors^8^ and thus CD163 and CD204 are useful markers for activation of TAMs in human samples. Furthermore, in malignant lymphoma, glioma, and kidney cancer, higher CD163 expression on TAMs is associated with worse clinical prognosis; however, no correlation exists between clinical prognosis and the number of CD204-expressing TAMs^9, 10^, CD204, also known as Class A scavenger receptor (SRA), has been shown to participate in the pathogenesis of atherosclerosis and the pattern recognition of pathogen infection^11^. CD163 is a hemoglobin scavenger receptor exclusively expressed in the monocyte-macrophage system. Furthermore, recent data indicate that soluble CD163 may be a valuable diagnostic parameter for monitoring macrophage activation in inflammatory conditions^12^.

Immune tolerance in the tumor microenvironment is closely involved in tumor progression caused by T-cell regulation via inhibitory signals of immune suppressive cytokine (IL-10), immune checkpoint molecules (programmed death-1 ligand 1 (PD-L1)), transforming growth factor-β, and prostaglandin E2^13^. PD-L1 is widely expressed by leukocytes and tumor cells, and a recent study demonstrated that PD-L1 is expressed on TAMs in almost all malignant lymphomas including adult T cell leukemia/lymphoma, follicular lymphoma, and diffuse large B-cell lymphoma^14, 15^.

In the present study, we investigated the localization of CD163- and CD204-positive cells in oral squamous cell carcinoma (OSCC). We also examined the levels of immune suppressive molecules produced by each TAM subset (CD163^+^CD204^+^, CD163^+^CD204^+^, and CD163^+^CD204^+^ TAMs) and association with clinical outcome.

## Materials and Methods

### Ethics Statement

The study design and methods were approved by the Institutional Review Board of Center for Clinical and Translational Research of Kyushu University Hospital (IRB serial number: 27–362). The methods were carried out in accordance with the approved guidelines. All patients or their relatives gave their informed consent within written treatment contract on admission and therefore prior to their inclusion in the study.

### Patients

We enrolled 46 patients with primary OSCC who were treated in the Department of Oral and Maxillofacial Surgery at Kyushu University Hospital from 2005 to 2015. The average age of the patients was 66.5 ± 10.3 years (range, 19–89). Twenty-seven patients were males and nineteen were females. Following the initial biopsy, all the specimens were fixed in 4% buffered formalin solution and embedded in paraffin blocks. The paraffin-embedded specimens were processed into 5 μm thick sections, stained with hematoxylin and eosin (HE) and examined by experienced oral pathologists to confirm the diagnosis and histologic grade. The tumor stage was classified according to the TNM classification of the International Union Against Cancer. Tumor histologic grade was defined according to the WHO classification. The mode of tumor invasion was determined from H&E stained specimens according to the Yamamoto-Kohama criteria as follows: grade 1 = well-defined borderline; grade 2 = cords, less-marked borderline; grade 3 = groups of cells, no distinct borderline; and grade 4 = diffuse invasion (4 C = cord-like type; 4D = widespread type). Patients and tumor characteristics are shown in Table 1.

**Table 1:** Association of tumor-associated macrophages (TAMs) with clinicopathologic characteristics in OSCC.

	Case (%)	CD163^+^ cells (/HPF)	*P*-value	CD204^+^ cells (/HPF)	*P*-value	CD163^+^CD204^+^ cells (/HPF)	*P*-value	CD25^+^ cells (/HPF)	*P*-value
Age^†^									
≤65	17 (37.0)	24.6 ± 15.2	*N*.*S*.	43.8 ± 28.1	*N*.*S*.	40.0 ± 28.4	*N*.*S*.	48.8 ± 28.5	*N*.*S*.
65<	29 (63.0)	27.8 ± 22.5		42.0 ± 23.1		38.0 ± 29.7		62.8 ± 31.6	
Gender^†^									
Male	27 (58.7)	30.7 ± 18.0	*N*.*S*.	49.1 ± 27.3	*N*.*S*.	43.6 ± 24.7	*N*.*S*.	58.2 ± 28.4	*N*.*S*.
Female	19 (41.3)	32.1 ± 23.6		44.7 ± 25.8		38.5 ± 22.2		49.5 ± 34.5	
Primary site^†^									
Tongue	22 (47.8)	28.4 ± 23.7	*N*.*S*.	41.8 ± 25.8	*N*.*S*.	34.7 ± 22.3	*N*.*S*.	56.2 ± 33.4	*N*.*S*.
Gingiva	16 (34.8)	35.2 ± 21.1		48.6 ± 29.2		45.8 ± 24.9		54.4 ± 30.3	
Buccal mucosa	6 (13.0)	32.6 ± 13.3		66.0 ± 22.8		57.5 ± 21.7		49.6 ± 25.1	
Oral floor	2 (4.3)	26.8 ± 3.6		42.0 ± 15.1		36.5 ± 12.0		57.6 ± 46.3	
Clinical stage*									
I	14 (30.0)	33.3 ± 28.1	*N*.*S*.	39.6 ± 25.7	*N*.*S*.	33.5 ± 23.6	*N*.*S*.	61.5 ± 27.4	0.024
II	16 (34.8)	25.3 ± 23.7		44.5 ± 26.1		36.7 ± 23.0		68.7 ± 21.8	r = −0.33
III	8 (17.4)	31.9 ± 11.3		51.5 ± 29.5		50.4 ± 20.4		35.8 ± 28.3	
IV	8 (17.4)	39.2 ± 23.3		61.5 ± 23.8		54.9 ± 21.5		37.5 ± 39.4	
T classification*									
T1	15 (32.6)	44.2 ± 22.7	*N*.*S*.	41.1 ± 30.6	0.017	33.5 ± 25.1	0.004	64.9 ± 27.5	0.018
T2	15 (32.6)	46.1 ± 23.4		43.0 ± 19.3	r = 0.34	36.6 ± 20.5	r = 0.40	63.1 ± 23.6	r = −0.34
T3	8 (17.4)	53.1 ± 14.3		48.7 ± 28.2		48.0 ± 17.9		37.3 ± 29.9	
T4	8 (17.4)	43.1 ± 19.5		66.5 ± 23.7		59.9 ± 21.6		37.8 ± 39.2	
Cervical nodal metastasis^†^									
+	9 (19.6)	48.8 ± 22.1	*N*.*S*.	49.5 ± 26.5	*N*.*S*.	47.8 ± 19.9	0.029	19.6 ± 32.8	*N*.*S*.
−	37 (80.4)	44.3 ± 20.1		46.9 ± 26.5		36.9 ± 25.1		26.4 ± 27.4	
Local recurrence^†^									
+	12 (26.1)	31.6 ± 16.4	*N*.*S*.	55.3 ± 23.4	*N*.*S*.	48.6 ± 22.5	*N*.*S*.	51.0 ± 26.4	*N*.*S*.
−	34 (73.9)	31.1 ± 22.5		44.6 ± 27.3		39.1 ± 23.5		56.1 ± 32.5	
Distant metastasis^†^									
+	5 (10.9)	49.9 ± 21.2	*N*.*S*.	78.2 ± 32.8	0.028	72.3 ± 30.8	0.023	25.5 ± 22.5	0.031
−	41 (89.1)	45.8 ± 21.1		43.6 ± 23.4		37.9 ± 19.7		58.4 ± 30.0	
Histological grade^†^									
Grade 1	31 (67.4)	21. 9 ± 18.7	*N*.*S*.	21.2 ± 26.6	*N*.*S*.	20.8 ± 24.1	*N*.*S*.	61.5 ± 27.7	*N*.*S*.
Grade 2	15 (32.6)	26.8 ± 20.5		28.2 ± 26.6		28.9 ± 20.2		40.9 ± 33.4	
Grade 3	0 (0.0)								
Mode of invasion* (YK criteria)									
Grade 1	3 (6.5)	33.0 ± 8.0	*N*.*S*.	15.2 ± 16.6	0.002	10.0 ± 14.7	0.001	62.8 ± 35.6	*N*.*S*.
Grade 2	7 (15.2)	45.8 ± 27.6		32.1 ± 15.5	r = 0.44	24.7 ± 16.0	r = 0.43	60.4 ± 29.3	
Grade 3	21 (45.7)	45.1 ± 22.1		49.7 ± 26.1		45.7 ± 20.1		57.4 ± 36.6	
Grade 4C	15 (32.6)	50.8 ± 12.5		53.8 ± 24.4		45.6 ± 21.6		46.8 ± 22.1	

*Spearman’s rank correlation coefficient, ^†^Mann-Whitney *U*-test and Wilcoxon signed-rank test. Not significant: *N*.*S*.

### Immunohistochemical analysis

After deparaffinization/hydration of sections, the sections were washed three times in TBST for 5 min each. The slides were boiled in 10 mM sodium citrate buffer, pH 6.0 and maintained at 121 °C for 10 min. The slides were cooled on the bench top for 30 min and washed in TBST three times for 5 min each. The sections were incubated in 3% H_2_O_2_ for 30 min and then washed in TBST three times for 5 min each. Sections were blocked with 100–400 µl blocking solution for 30 min at room temperature, followed by incubation with primary antibody overnight at 4 °C. We used mouse anti-CD163 (Clone 10D6; Novocastra, Newcastle, UK, 1:400 dilution), mouse anti-CD204 (Clone SRA-E5; Transgenic, Kumamoto, Japan, 1:200 dilution), rabbit anti-CD25 (Clone ab128955; Abcam, Cambridge, UK 1:200 dilution), rabbit anti-IL-10 (Clone ab34843; Abcam, 1:50 dilution), rabbit anti-PD-L1 (E1L3N; Cell Signaling Technology, USA, 1:200 dilution), and goat anti-CD69 (clone H-20; SANTA CRUZ, Heidelberg, Germany 1:50 dilution). Antibody was removed and 100–400 µl DAB (Peroxidase Stain DAB Kit®, Nacalai Tesque, Japan) was added to each section. We performed counterstaining with hematoxylin and washed the sections in dH_2_O two times for 5 min each. After dehydration, we mounted the sections with coverslips.

### Double immunofluorescence analysis

We first incubated sections with Blocking Buffer for 60 min and then incubated the sections with primary antibodies (as listed above). Apply these antibodies and the respective groups were CD163 IL-10, CD163 PD-L1, CD204 IL-10, CD204 PD-L1 and incubated for 3 h at room temperature. We rinsed the samples three times in TBST for 5 min each and then incubated the samples in fluorochrome-conjugated secondary antibody (Alexa Fluor® 594; Thermo Fisher Scientific, Waltham, MA, USA) diluted in Antibody Dilution Buffer for 1–2 h at room temperature in the dark. We rinsed the samples in TBST Coverslip slides with DAPI (Vectashield with DAPI®; VECTOR LABORATORIES, USA). About double staining of CD163 and CD204 was same of host animal so that we labeled FITC (Fluorescein Labelling Kit-NH2®; Dojindo Laboratories, Kumamoto, Japan).

### Evaluation of macrophages and activated T cells

The numbers of CD25, CD163 and CD204 positive cells in immunohistochemical staining were counted in 4 mm^2^ sections from five independent high-power microscopic fields (400×; 0.0625 μm^2^) of cancer nest in immunohistochemistry. The numbers of CD163^+^CD204^+^ cells in double immunofluorescence staining were counted in the same way.

### Culture and purification of TAMs

PBMCs (5 × 10^5^ cells/ml) from the OSCC patients were cultured in PBS and stimulated with PMA 40 ng/ml (phorbol 12-myristate 13-acetate; Wako, Tokyo, Japan) and ionomycin 4 μg/ml (Ionomycin Calcium; Wako) for 6 h. CD163- and CD204-positive macrophages were isolated from the cultured PBMCs by positive selection with magnetic beads (PE or FITC Microbeads; Miltenyi Biotec Inc., Auburn, CA) according to the manufacturer’s manual. CD3^+^ T cells were purified from PBMCs without culture by negative selection with magnetic beads (CD3^+^ Microbeads, Miltenyi Biotec Inc.).

### Co-culture of TAMs and CD3^+^ T cells

CD3^+^ T cells (5 × 10^5^ cells/ml) were pre-incubated for 5 days in complete COSMEDIUM 006X® medium supplemented with 5% autoserum, and then pre-incubated for 6 h in PBS for additional PMA and ionomycin. These activated CD3^+^ T cells (5 × 10^5^ cells/ml) co-cultured with TAMs (5 × 10^5^ cells/ml) for 3 days in Lymphocyte Preservation Assist® (Takara Bio, Otsu, Japan) with 5% autoserum. The cells were examined under the microscope or harvested for flow cytometric analysis for expression of surface markers. In some cases, cell cultures were carried out on glass slides for observation under confocal microscopy.

### Flow cytometric analysis

Harvested cells were washed with PBS supplemented with 1% BSA. After washing, the cells were incubated at room temperature for 20 min with PE anti-human CD163 antibodies (Clone GHI/61, IgG_1,κ_; BioLegend, San Diego, CA, USA), FITC anti-human CD204 antibodies (Clone REA460, IgG_1_; Milteni Biotec, Bergisch Gladbach, Germany), APC anti-human PD-L1 (Clone 29E.2A3, IgG2_b,κ_; BioLegend), and PerCP/Cy5.5 anti-human IL-10 (Clone JES3–9D7, IgG_1,κ_; BioLegend). PE mouse IgG_1,κ_ (BioLegend), FITC REA Control antibodies IgG (Milteni Biotec), APC mouse IgG_2b,κ_ (BioLegend), PerCP/Cy5.5 Rat IgG_1,κ_ (BioLegend) and APC mouse IgG_1,κ_ (BioKegend) were used as negative control antibodies. CD3^+^ T cells and TAMs in co-culture were analyzed using gating for CD3^+^, CD163^+^ CD204^+^ or CD163^−^CD204^+^ cells, respectively. Apoptosis of CD3^+^ T cells following culture with TAMs was measured by staining with 7-aminoactinomycin D (7-ADD) (BioLegend). Activation of CD3^+^ T cells following culture with TAMs was measured by staining with APC anti-human CD69 antibodies (Clone FN50, IgG1; BioLegend).

### Statistical analysis

All statistical analyses were performed by JMP software version 11 (SAS Institute, NC, USA). Mann–Whitney U test, Wilcoxon test and Spearman’s rank correlation coefficient was used to assess the significant differences between each group. Progression-free survival and overall survival were estimated by the Kaplan–Meier method and curve comparisons were calculated using the log-rank test. In all analyses, *P* values ≤ 0.05 were considered statistically significant.

## Results

### Expression of TAM markers in OSCC

**Figure 1:**
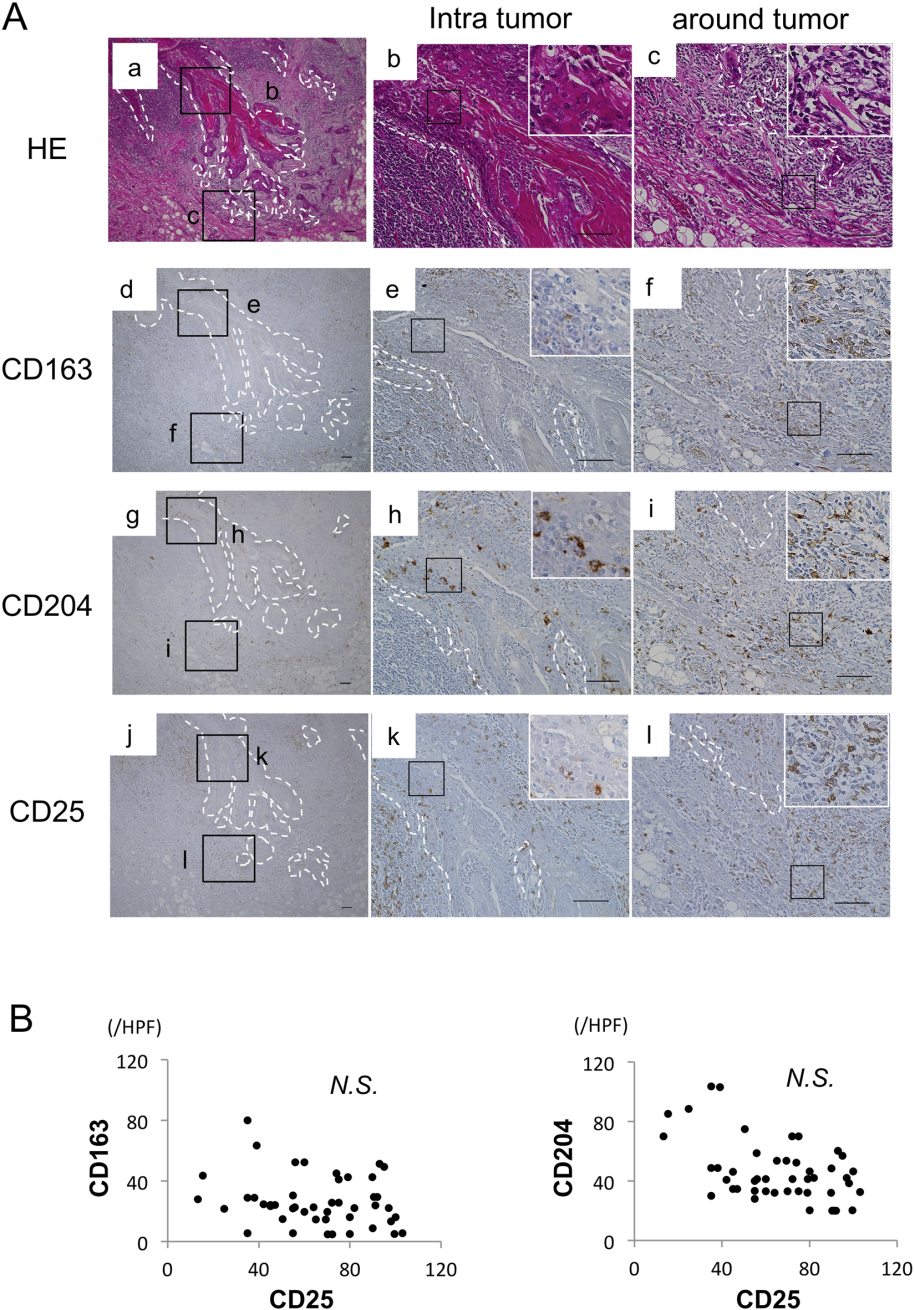
Correlation between tumor-associated macrophages (TAMs) and activated immune cells in OSCC patients. (**A**) Representative images of paraffin sections in/around tumor stained with H&E (a–c), CD25 (j–l), CD163 (d–f) and CD204 (g–i) antibodies (brown). Counterstaining with Mayer’s hematoxylin is shown in blue. Scale bars, 100 μm. (**B**) Correlation between the number of CD163^+^ or CD204^+^ and CD25^+^ cells in 46 OSCC patients. Statistically significant differences between groups were determined by Spearman’s rank correlation.

**Figure 2:**
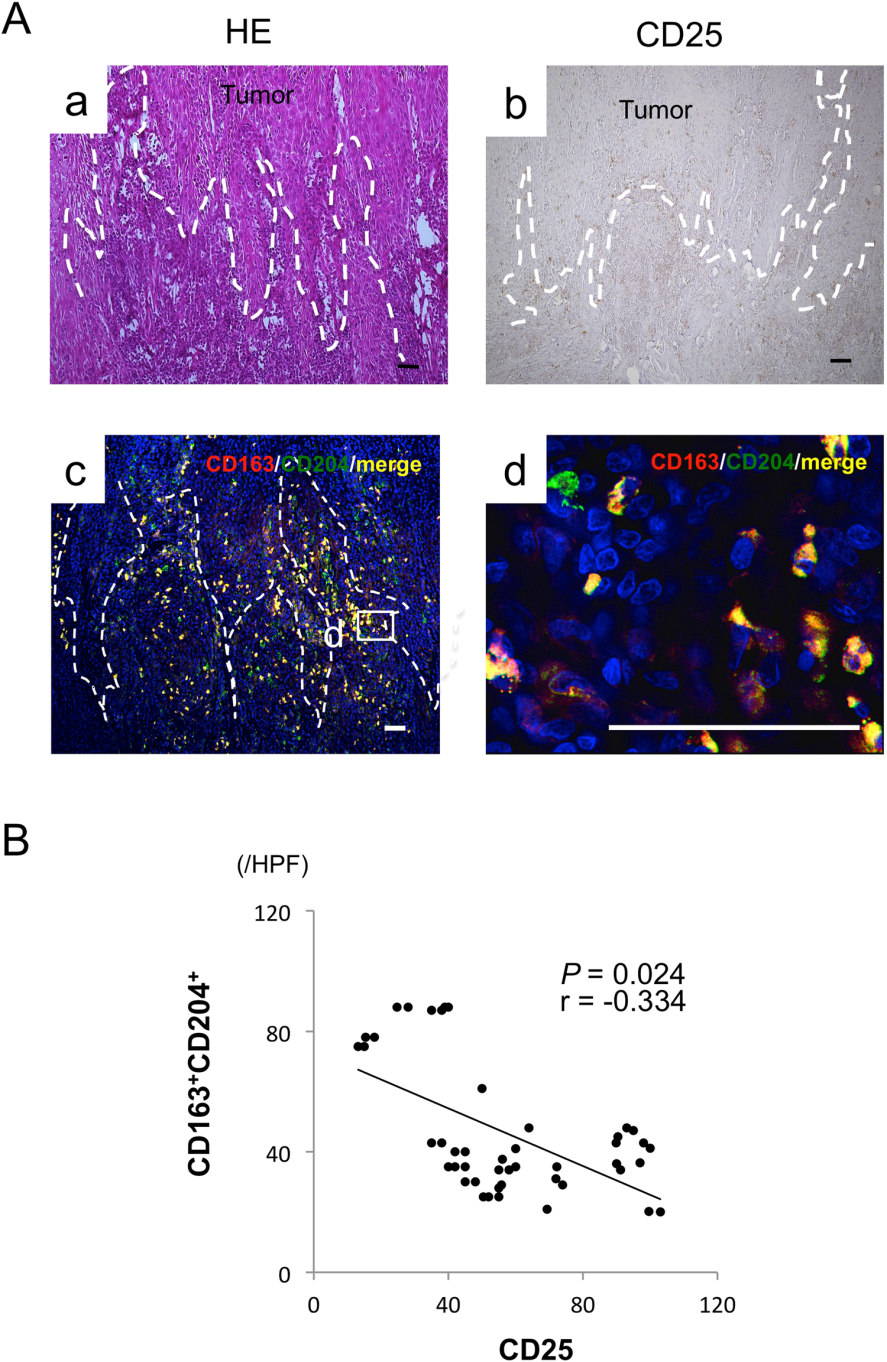
Co-localization of TAM markers in OSCC patients. (**A**) Representative images of paraffin sections in/around tumor stained with H&E (a) and CD25 (b) antibodies (brown). Counterstaining with Mayer’s hematoxylin is shown in blue. Double immunofluorescence staining performed with CD163 (red), CD204 (green), and DAPI for staining nuclei (blue) at low (c) and high magnification (d). (c,d) Merged CD163 and CD204 images (yellow). Scale bars, 50 μm. (**B**) Correlation between the number of CD163^+^CD204^+^ and CD25^+^ cells in 46 OSCC patients. Statistically significant differences between groups were determined by Spearman’s rank correlation.

We first performed immunohistochemical staining to evaluate the distribution of TAMs (CD163, CD204) and activated immune cell markers (CD25 and CD69) in OSCC tissues. Expression of CD163 was detected in tumor stroma and around tumors, while that of CD204 was strongly detected in/around tumors. Expression of CD25 was diffusely detected in/around tumors (Fig. 1A). The number of CD163- and CD204-positive cells did not correlate with that of CD25-positive cells (Fig. 1B). Moreover, double immunofluorescence analysis found that CD163^+^CD204^+^ cells were frequently detected around tumors (Fig. 2A) and showed a correlation with the number of CD25-positive cells (r = −0.445; *P* < 0.05) (Fig. 2B) and CD69-positive cells (r = −0.359; *P* < 0.05) (Supp. Fig. 1).

### Co-localization of TAM markers and immunosuppressive molecules in OSCC

**Figure 3:**
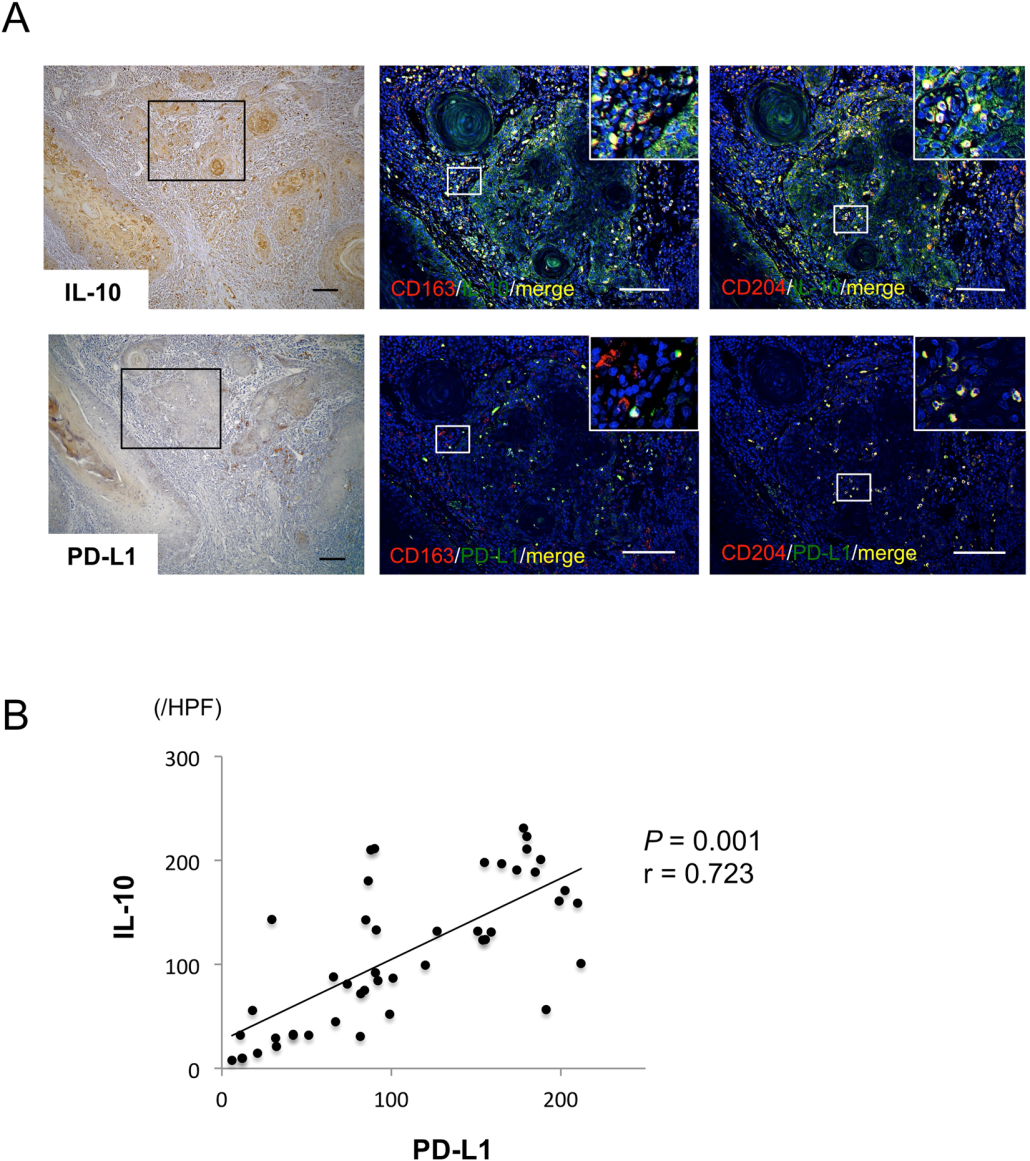
Co-localization of TAM markers and immunosuppressive molecules in OSCC. Representative images of paraffin sections in/around tumor stained with IL-10 (a) and PD-L1 (d). Counterstaining with Mayer’s hematoxylin is shown in blue. Double immunofluorescence staining performed with TAM markers (red) such as CD163 (b,e) and CD204 (c,f), and immunosuppressive molecules (green) such as IL-10 (b,c) and PD-L1 (e,f), and DAPI for staining nuclei (blue). (b,c,e,f) Merged CD163 and CD204 images (yellow). Scale bars, 50 μm.

To clarify whether TAMs express immunosuppressive molecules, double immunofluorescence staining with TAM markers and IL-10 or PD-L1 was performed. As shown in Fig. 3A, CD163- and CD204-positive cells (red) were co-localized with IL-10- and PD-L1-positive cells (green). Moreover, number of PD-L1-positive cells was positively correlated with that of IL-10-positive cells (Fig. 3B). These results suggest that TAMs might suppress the immune response to OSCC through increased production of IL-10 and PD-L1.

### Expression levels of immunosuppressive molecules produced by TAMs

**Figure 4:**
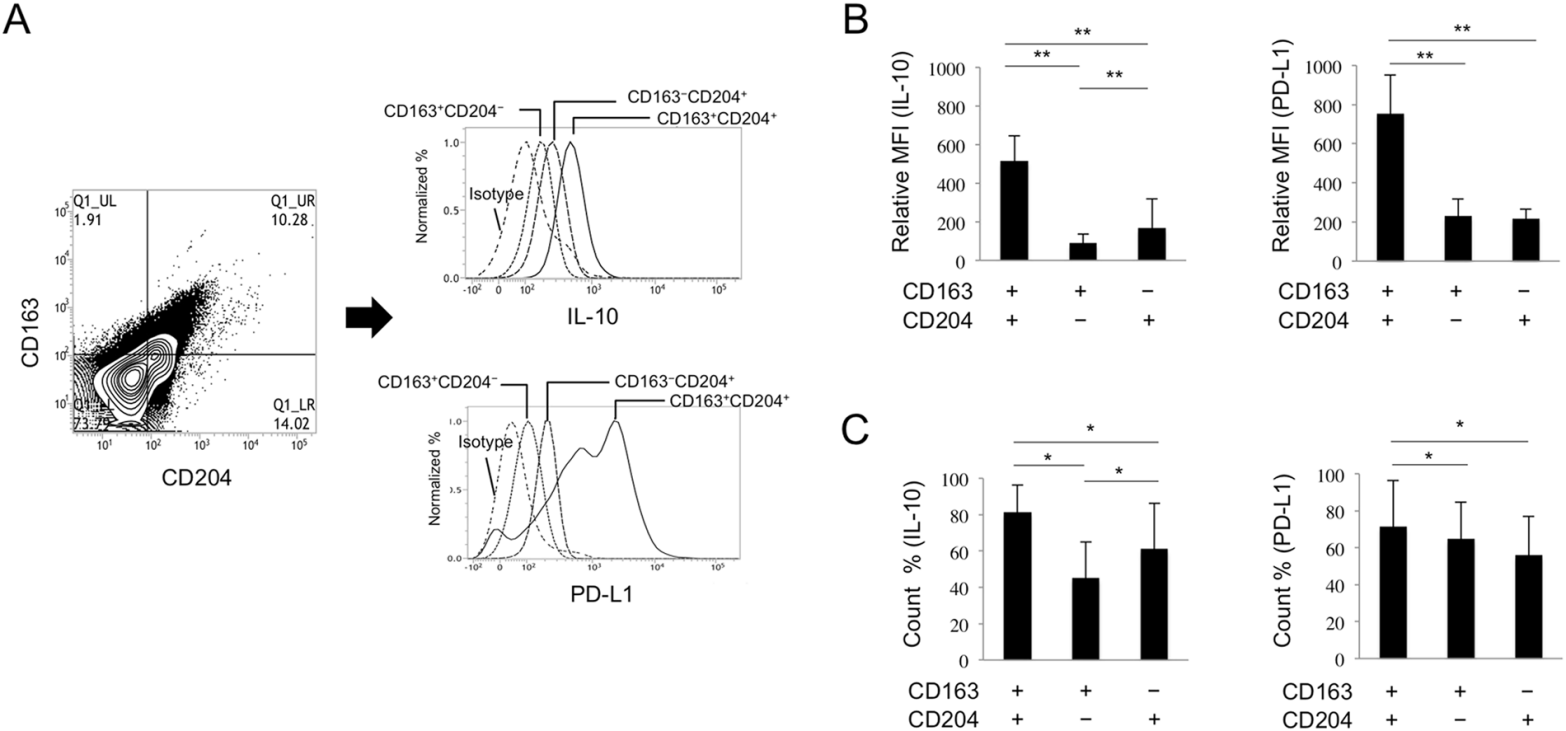
IL-10 and PD-L1 expression on TAM subsets in OSSC patients. (**A**) Flow cytometric analysis of IL-10 and PD-L1 expression on cultured TAM subsets. The detailed methods for cultivating cells are described in the Materials and Methods section. (**B**) IL-10 and PD-L1 expression (MFI; mean fluorescent intensity) on TAM subsets and (**C**) the number of IL-10^+^ and PD-L1^+^ cells were tested using flow cytometry (n = 3 for each subset). Statistically significant differences between groups were determined by Wilcoxon signed-rank test (**P < 0.01, *P < 0.05).

As IL-10 and PD-L1 were expressed by both CD163- and CD204-positive cells, we next compared the expression levels of these molecules produced by each TAM subset (CD163^+^CD204^−^, CD163^−^CD204^+^, and CD163^+^CD204^+^ cells) in PBMCs from OSCC patients as described in the Materials and Methods. As shown in Fig. 4 and Supp. Fig. 2, we found that CD163^+^CD204^+^ cells expressed higher levels and numbers of IL-10 and PD-L1 on the cell surface in comparison with CD163^+^CD204^−^ and CD163^−^CD204^+^cells, indicating a difference in the expression of immunosuppressive molecules among the TAM subsets.

### Effects of apoptotic and activation status on CD3^+^ T cells by co-culture with TAMs

**Figure 5:**
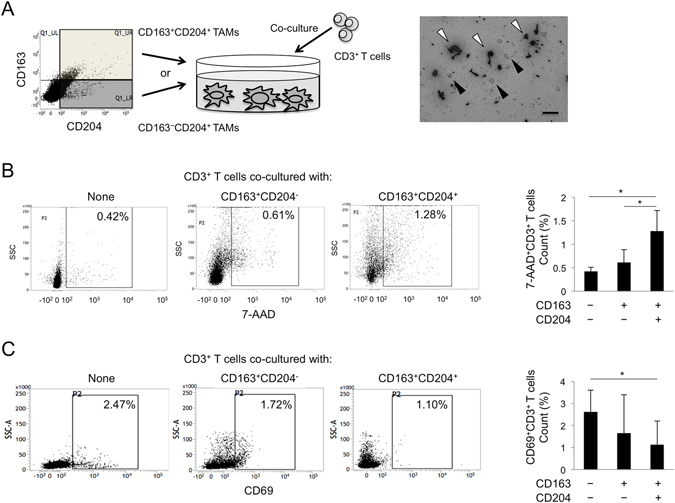
T cell regulation and apoptosis by co-culture with TAM subsets. (**A**) Scheme and representative image for the co-culture of TAM subsets (white arrowhead) and CD3^+^ T cells (black arrowhead) for 5 days. The detailed methods for cultivating cells are described in the Materials and methods section. Scale bars, 10 μm. (**B**) The population of 7-AAD^+^CD3^+^ T cells co-cultured with TAM subsets from a representative OSCC patient. The number of 7-AAD^+^CD3^+^ T cells co-cultured with TAM subsets was analyzed using flow cytometry (n = 3 for each subset). Statistically significant differences between groups were determined by Wilcoxon signed-rank test (*P < 0.05). (**C**) The population of CD69^+^CD3^+^ T cells co-cultured with TAM subsets from a representative OSCC patient. The number of CD69^+^CD3^+^ T cells co-cultured with TAM subsets was analyzed using flow cytometry (n = 3 for each subset). Statistically significant differences between groups were determined by Wilcoxon signed-rank test (*P < 0.05).

To quantitatively confirm the immune suppression inducing effect on TAMs, we performed flow cytometric analysis for purified CD3^+^ T cells co-cultured with purified CD163^−^CD204^+^ or CD163^+^CD204^+^ cells as described in the Materials and Methods (Fig. 5A). The number of purified CD163^+^CD204^−^ cells was too small to measure the appropriate cell count by flow cytometry. The dot plots allowed us to distinguish dead CD3^+^ T cells from viable cells and TAMs. The ability of TAMs to cause apoptosis of CD3^+^ T cells was further substantiated by measuring 7-AAD^+^CD3^+^ T cells following their co-culture with purified CD163^−^CD204^+^ or CD163^+^CD204^+^ cells. The number of dead CD3^+^ T cells after co-culture with CD163^+^CD204^+^ was significantly higher than that after co-culture with CD163^−^CD204^+^ cells and without TAMs (Fig. 5B). On the other hand, the ability of TAMs to inhibit activation of CD3^+^ T cells was further substantiated by measuring CD69^+^CD3^+^ T cells following their co-culture with purified CD163^−^CD204^+^ or CD163^+^CD204^+^ cells. The number of activated CD3^+^ T cells after co-culture with CD163^+^CD204^+^ was significantly lower than that after co-culture with CD163^−^CD204^+^ cells and without TAMs (Fig. 5C).

### Associations of TAMs with clinical and pathological findings of OSCC patients

We next examined the associations of TAMs with the clinicopathologic factors of OSCC patients. TAMs were counted by the following three methods: (1) CD163^+^ cells detected by single staining, (2) CD204^+^ cells detected by single staining, and (3) CD163^+^CD204^+^ cells detected by double staining. As shown in Table 1, the number of CD163^+^ cells did not show significant differences among all of the clinicopathologic factors, while that the numbers of CD204^+^ and CD163^+^CD204^+^ cells were positively correlated with clinical T classification, mode of invasion, and the prevalence of distant metastasis. Interestingly, the OSCC patients with cervical nodal metastasis showed a significant increase only in the number of CD163^+^CD204^+^ cells. On the other hand, the number of CD25^+^ cells was negatively correlated with clinical stage, clinical T classification, and the prevalence of distant metastasis.

### Associations of TAMs with clinical outcomes and prognosis among the groups

**Figure 6:**
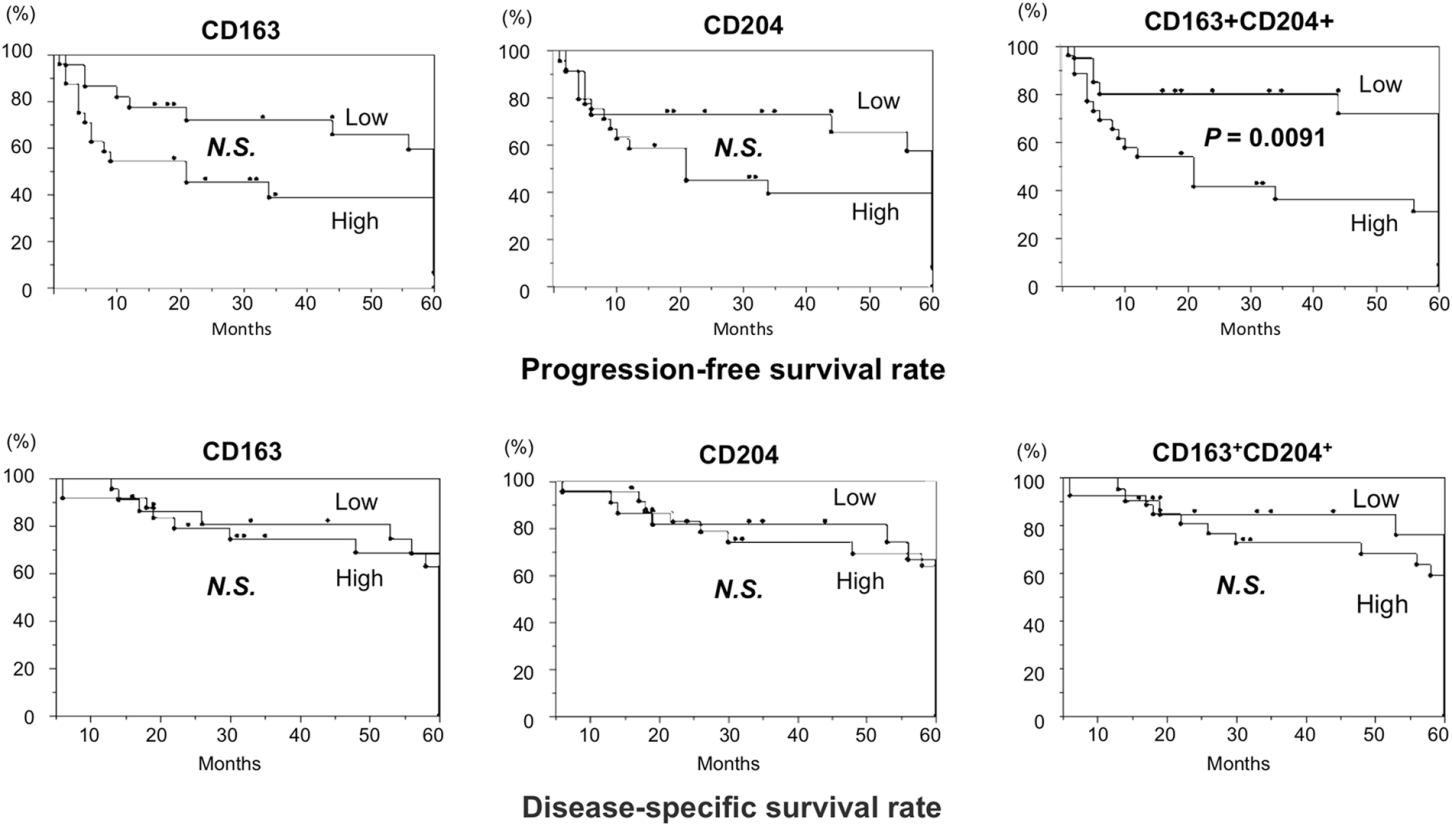
Survival curves according to the expression of TAM subsets in OSCC. The survival rates were calculated by the Kaplan-Meier method with high versus low expression of CD163^+^, CD204^+^, or CD163^+^CD204^+^ TAMs. The classifications are described in the Materials and Methods section. Statistically significant differences between groups were determined by log-rank test.

To evaluate the correlation between TAMs and the clinical outcomes of OSCC patients, the survival rates were calculated by the Kaplan-Meier method. The OSCC patients were divided into two groups according to the mean number of TAMs (CD163^+^, CD204^+^, and CD163^+^CD204^+^ cells): low and high expression groups. In the progression-free survival curves, the patients in the high CD163^+^CD204^+^ expression group had a significantly more unfavorable outcome than those in the low expression group, while had no significant difference among the groups of CD163^+^and CD204^+^ cells. In the disease-specific survival curves, the patients had no significant difference among the groups of CD163^+^, CD204^+^, and CD163^+^CD204^+^ cells (Fig. 6). Univariate analysis revealed that progression-free survival was associated with advanced age, YK criteria, number of CD163^+^CD204^+^ cells (Table 2). Multivariate analysis identified number of CD163^+^CD204^+^ cells as a marginally significant prognostic factor for progression-free survival (hazard ratio, 1.97; P = 0.0722) (Table 2).

**Table 2:** Univariate and multivariate analysis of progression-free survival and disease-specific survival in advanced stage.

**Progression-free survival**					
Variables	Categories	Univariate analysis		Multivariate analysis	
		Progression-free survival		Progression-free survival	
		HR (95% CI)	*P*	HR (95% CI)	*P*
Age (y)	<65 vs z 65	3.46 (1.01–13.35)	0.05*		
Pathologic tumor status	pT1 +pT2 vs pT3 +pT4	2.00 (0.57–7.07)	0.27		
Pathologic node status	pN− vs pN+	0.37 (0.05–1.81)	0.23		
Pathologic metastasis status	pM0 vs pM1	2.60 (0.39–21.47)	0.32		
Clinical stage	1 vs 2 + 3 + 4	1.04 (0.28–4.09)	0.95		
Histological grade	1 vs 2	1.59 (0.45–5.65)	0.47		
YK criteria	1 +2 vs 3 + 4	8.05 (1.31–156.4)	0.02*		
CD163 positive cells	Low vs high	1.53 (0.77 – 3.02)	0.21	1.18 (0.57 – 2.42)	0.64
CD204 positive cells	Low vs high	1.79 (0.92 – 3.52)	0.08	1.77 (0.89 – 3.52)	0.10
CD163^+^CD204^+^ cells	Low vs high	2.07 (1.04–4.32)	0.03*	1.97 (0.94 – 4.30)	0.07
**Disease-specific survival**					
**Variables**	**Categories**	**Univariate analysis**		**Multivariate analysis**	
		**Disease-specific survival**		**Disease-specific survival**	
		**HR (95% CI)**	***P***	**HR (95% CI)**	***P***
Age (y)	<65 vs z65	1.39 (0.69–2.86)	0.34		
Pathologic tumor status	pT1 +pT2 vs pT3 +pT4	1.79 (0.84–3.63)	0.12		
Pathologic node status	pN− vs pN+	0.99 (0.39–2.17)	0.98		
Pathologic metastasis status	pM0 vs pM1	5.58 (1.17–21.3)	0.03		
Clinical stage	1 vs 2 + 3 + 4	1.18 (0.57–2.54)	0.65		
Histological grade	1 vs 2	1.20 (0.58–2.41)	0.60		
YK criteria	1 +2 vs 3 + 4	1.23 (0.54–3.32)	0.62		
CD163 positive cells	Low vs high	1.22 (0.57–2.50)	0.58	1.20 (0.56–2.46)	0.61
CD204 positive cells	Low vs high	1.46 (0.71–2.94)	0.28	1.16 (0.41–4.14)	0.79
CD163^+^CD204^+^ cells	Low vs high	1.45 (0.72–2.97)	0.29	1.22 (0.34–3.41)	0.72

HR, hazard ratio; CI, confidence interval, *Significant.

These results suggest that CD163^+^CD204^+^ cells play a critical role in the suppression of tumor immunity and are involved in the invasion and metastasis in OSCC.

## Discussion

In 1908, Mechnikov *et al*. first described that macrophages were professional phagocytes and play key roles in inflammation and natural cellular immunity^16^. In 1970’s, many researchers thought that activated macrophages were important effector cells in cytotoxic killing of tumor cells^17^. Macrophages are classified into two major subsets: classically activated (M1) macrophages stimulated by Th1-type responses and alternatively activated (M2) macrophages stimulated by Th2-type responses^6, 7^. M1 macrophages secrete pro-inflammatory cytokines and contribute to microbicidal and tumoricidal immunity, whereas M2 macrophages scavenge debris and contribute to angiogenesis, suppression of adaptive immunity, wound healing and fibrosis by producing IL-10 and CCL18. Pollard *et al*.^18^ reported that macrophages infiltrating cancer tissues polarized to the M2 phenotype and were involved in the development of the tumor microenvironment by inducing angiogenesis and immune suppression. Recent studies have referred to these M2-polarized macrophages as TAMs, which express specific markers such as CD163 and CD204^19, 20^.

CD163 is a member of the scavenger receptor cysteine-rich family class B and is expressed on most subpopulations of mature tissue macrophages^21^. The best characterized function of CD163 is essentially a homeostatic one and is related to the binding of hemoglobin:haptoglobin complexes. Furthermore, CD163-positive macrophages play a role in the resolution of inflammation, as they are found in high numbers in inflamed tissue^22, 23^. We found the strong infiltration of CD163-positive cells but not CD204-positive cells in the lesions of non-specific ulcer (Supp. Fig. 3). Komohara *et al*. indicated that CD163 antigen might be a better marker of the M2 anti-inflammatory phenotype in clear cell renal cell carcinoma tissues compared with CD204.

CD204 is a prototypic member of a family of structurally diverse transmembrane receptors collectively termed scavenger receptors^24^. CD204 is preferentially expressed in dendritic cells and macrophages. CD204 functions as a pattern recognition receptor capable of binding a broad range of ligands, including chemically modified or altered molecules, bacterial surface components, and apoptotic cells, and plays roles in lipid metabolism, atherogenesis, and metabolic processes^25, 26^. Several studies have shown that CD204 deficiency resulted in impaired protection against pathogen infection^27, 28^, which has been partially attributed to the increased susceptibility of CD204-deficient animals to the overproduction of pro-inflammatory cytokines during endotoxic shock^29^. Emerging evidence also implicates CD204 as a suppressor in the inflammatory response^30, 31^. Furthermore, in esophageal squamous cell carcinomas, CD204 was demonstrated as a better marker for the TAM populations associating with tumor progression compared with CD163^32^. Thus, there is not yet a consensus regarding which of the two markers are more suitable for TAMs.

In the present study, we examined the expression and immunosuppression of TAMs in OSCC using both CD163 and CD204. There were clear differences in the localization of CD163- and CD204-positive cells in OSCC sections. Interestingly, the double immunofluorescent staining data revealed that CD163^+^CD204^+^TAMs mainly infiltrated around tumors and expressed higher levels of IL-10 and PD-L1 compared with other TAM subsets. Moreover, there is a positive correlation between number of IL-10 and PD-L1. IL-10 leads to the phosphorylation of STAT3 and then IL-10/STAT3 signaling induces PD-L1 overexpression^33^. We previously reported that phosphorylated-STAT3 expression was localized in the nucleus of the cancer cells and scattered widely in the cancer nests from patients with OSCC at advanced clinical stage^34^. In addition, CD163^+^CD204^+^ TAMs were also found to activate the function of T cell regulation and apoptosis. These findings indicate that CD163^+^CD204^+^ TAMs might play an important role in immune suppression and tumor progression via IL-10-Stat3-PD-L1 signaling.

Recently, PD-1 ligand 2 (PD-L2) was identified as a second ligand for PD-1. PD-L1 constitutively expressed by various immune cells including T cells, B cells, macrophages, DCs, and tumor cells. On the other hands, PD-L2 expression is limited to macrophages and DCs^35^. In malignant tumor, TAMs suppressed anti-tumor immune responses by overexpression of PD-L1/2^36^. Therefore, further examinations are required to elucidate the expression of PD-L2 in each TAM subsets in OSCC.

We next examined the association of the TAMs with the prognosis of OSCC patients. The number of CD204^+^ and CD163^+^CD204^+^ TAMs in OSCCs was positively correlated with clinical T classification and distant metastasis. Moreover, the numbers of CD163^+^CD204^+^ TAMs also significantly associated with the progression-free survival curves. However, the association between the expression of TAM markers and clinical prognosis for cancer patients remains controversial. Hirayama *et al*.^37^ reported that a high number of CD204^+^ TAMs in lung SCCs was significantly correlated with advanced clinicopathological parameters and poor prognosis. Another study showed that there was no significant difference in clinicopathological factors and clinical prognosis between high and low expression groups of CD163^+^ TAMs in OSCC^38^. These results were consistent with our results in the present study. On the contrary, in gliomas, both CD204^+^ and CD163^+^ TAMs were positively correlated with the histological malignancy^39^. This contradictory conclusion may be due to different tumor types or methodologies.

In conclusion, we have confirmed that CD163^+^CD204^+^ TAMs promote T-cell apoptosis and immunosuppression via IL-10 and PD-L1 and predict unfavorable prognosis in OSCC patients. We are currently examining which of the TAM subsets contribute to the tumor proliferation and angiogenesis in OSCC. A more thorough understanding of the role of each TAM subset could lead to the development of novel pharmacological strategies aimed at disrupting TAMs or their products and inhibiting the progression and/or metastasis of OSCC.

## Electronic supplementary material

Supplementary Figure 1-3
