# Supplementary material for: CD163+CD204+ tumor-associated macrophages contribute to T cell regulation via interleukin-10 and PD-L1 production in oral squamous cell carcinoma
Source: Sci Rep. 2017 May 11;7:1755. doi: 10.1038/s41598-017-01661-z (PMC5431876; doi:10.1038/s41598-017-01661-z)
Supplement: Supplementary file 1 — Supplementary Figure 1-3 [file 41598_2017_1661_MOESM1_ESM.pdf]

CD163^{+}CD204^{+} tumor-associated macrophages contribute to T cell regulation via interleukin-10 and PD-L1 production in oral squamous cell carcinoma

Keigo Kubota, DDS[1, 4], Masafumi Moriyama, DDS, PhD[1, 2, 4*], Sachiko Furukawa, DDS, PhD[1], Haque A S M Rafiul, DDS[1], Yasuyuki Maruse, DDS, PhD[1], Teppei Jinno, DDS, PhD[1], Akihiko Tanaka, DDS, PhD[1], Miho Ohta, DDS, PhD[1], Noriko Ishiguro, DDS[1], Masaaki Yamauchi, DDS[1], Mizuki Sakamoto DDS[1], Takashi Maehara, DDS, PhD[1], Jun-Nosuke Hayashida, DDS, PhD[1], Shintaro Kawano, DDS, PhD[1], Tamotu Kiyosima, DDS, PhD[3], and Seiji Nakamura, DDS, PhD[1]

[1] Section of Oral and Maxillofacial Oncology, Division of Maxillofacial Diagnostic and Surgical Sciences, Faculty of Dental Science, Kyushu University, Fukuoka, 812-8582, Japan

[2] OBT Research Center, Faculty of Dental Science, Kyushu University, 812-8582, Japan

[3] Laboratory of Oral Pathology, Division of Maxillofacial Diagnostic and Surgical Sciences, Faculty of Dental Science, Kyushu University, Fukuoka, 812-8582, Japan

[4] These authors contributed equally to this work.

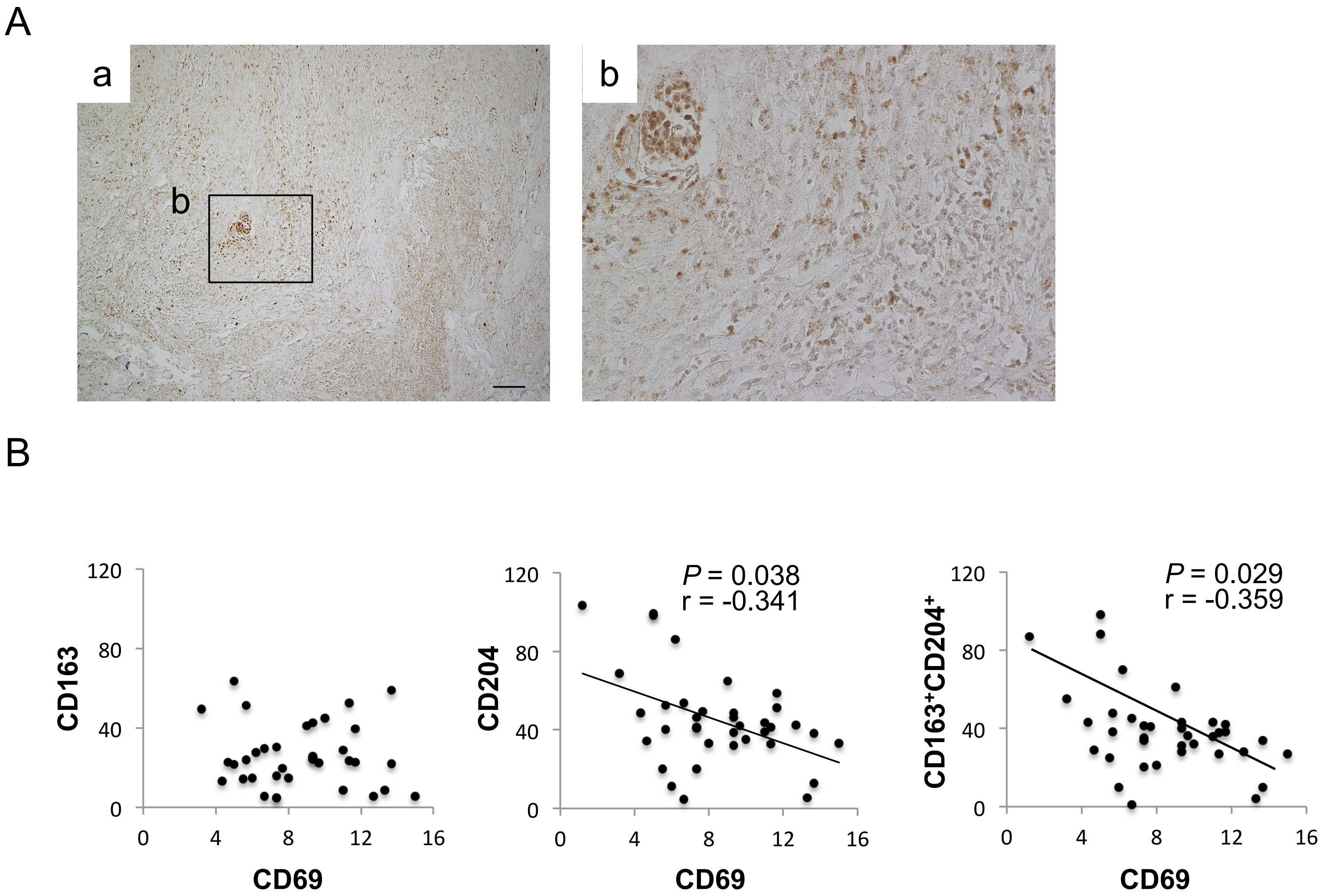

Supplementary Figure 1. Expression and localization of TAMs in OSCC patients. (A) Representative images of paraffin sections around tumor stained with CD69 antibodies (brown) Counterstaining with Mayer's hematoxylin is shown in blue. Scale bars, 100 μm. (B) Correlation between the number of $CD163^{+}$, $CD204^{+}$ or $CD163^{+}CD204^{+}$ and $CD69^{+}$ cells in 36 OSCC patients. Statistically significant differences between groups were determined by Spearman's rank correlation.

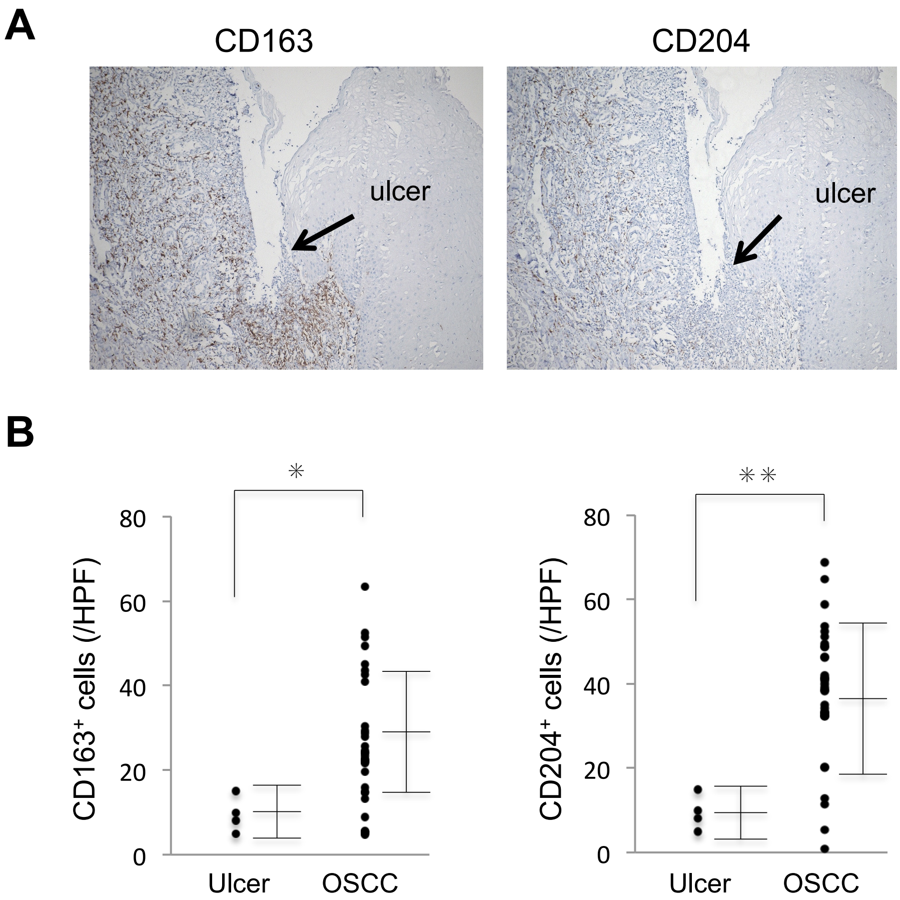

Supplementary Figure 2. Expression and localization of TAMs in non-specific ulcer patients. (A) Representative images of paraffin sections in/around tumor stained with CD163 and CD204 antibodies (brown) Counterstaining with Mayer's hematoxylin is shown in blue. Scale bars, 100 μm. (B) The number of CD163+ and CD204+ cells in patients with non-specific ulcer (n=5) and OSCC (n=46) was calculated by immunohistochemical staining. Statistically significant differences between groups were determined by Mann–Whitney *U* test (***P* < 0.01, **P* < 0.05).

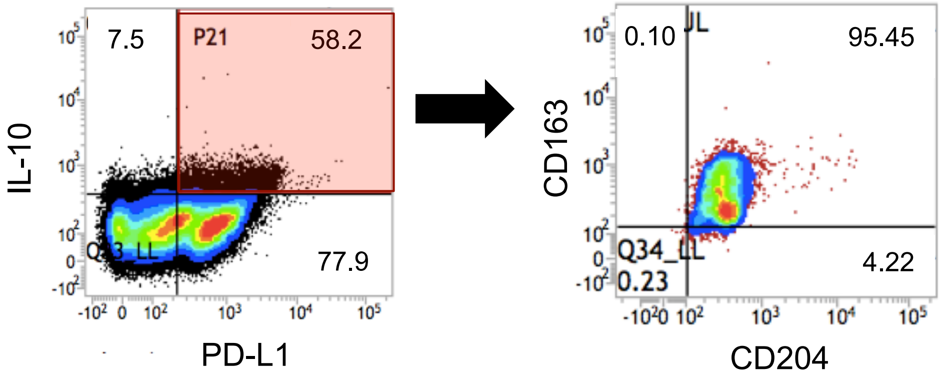

Supplementary Figure 3. IL-10 and PD-L1 expression on TAM subsets in patients with OSSC**.** This figure showed that changed the gating methods from Figure 4. Flow cytometric analysis of IL-10 and PD-L1 expression on cultured TAM subsets. The detailed methods for cultivating cells are described in the Materials and methods section.
